# Multiple Exposures of Plasma to Nanoparticles: A Novel Tool to Personalize Biomolecular Coronas and Fractionate Fluids

**DOI:** 10.1021/acs.analchem.4c05573

**Published:** 2025-06-30

**Authors:** Alberto Martinez-Serra, Jack Cheeseman, Asia Saorin, Mahmoud G. Soliman, Marko Dobricic, Daniel I. R. Spencer, Marco P. Monopoli

**Affiliations:** a Chemistry Department, 8863 Royal College of Surgeons in Ireland (RCSI), 123 St. Stephen’s Green, Dublin 2, Ireland; b 172254 Ludger Ltd., Culham Campus, Abingdon OX14 3EB, Oxfordshire, United Kingdom

## Abstract

Nanoparticles (NPs) have emerged as a valuable tool for biomarker discovery due to their ability to interact with biological fluids and form biomolecular coronas. In this study, we introduce a multiple exposure method that uses NPs to fractionate biological fluids and obtain personalized coronas. By repeatedly exposing plasma to silica NPs, we observed a progressive change of the biomolecule profile in both the pellet and supernatant. The varying protein and glycan composition of the corona was characterized using techniques such as SDS–PAGE, mass spectrometry, and UHPLC. Notably, the corona’s composition evolved with each exposure cycle, reflecting the selective binding of proteins and glycosylated molecules from a corona of high-affinity biomolecules to a more diverse corona with very distinct structures. By tracing the sequential modification of protein and glycan composition, we believe that the method can be useful to trace specific biomarker profiles, offering a noninvasive alternative to conventional diagnostic processes with the potential to become a useful tool for disease monitoring and advanced biomedical applications.

## Graphical abstract

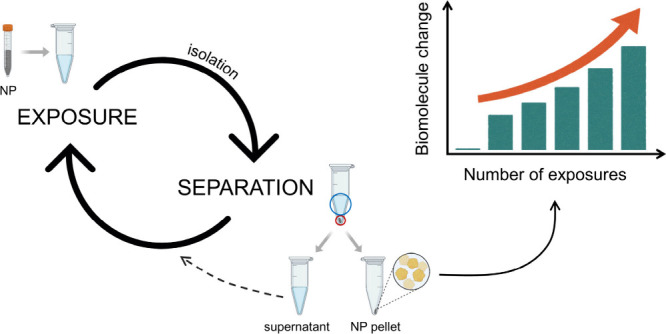

## Introduction

Blood plasma has been used during the past decade as a relevant environment to study the biological interactions of nanoparticles (NPs) due to the rich diversity of biomolecules and its capacity to act as a repository for biomarkers.^1–4^ According to the HUPO Plasma Proteome Project, blood plasma contains over 3000 proteins, with a dynamic range of abundance across 12 orders of magnitude.^5^ In addition, plasma proteome is made up for almost 99% in mass by 22 abundant proteins (e.g., albumin, IgG, fibrinogen, etc.), while the remaining 1% contains the remaining proteins, including the classical plasma proteins that are largely secreted by the liver and intestines but also tissue leakage proteins, interleukines, etc.^6^ While the plasma proteome contains many proteins of potential biomarker relevance, as they often signal early disease processes,^7^ the complexity to trace them due to the high abundance of certain proteins, such as albumin, makes the detection of low-abundance biomarkers challenging. These less abundant proteins are also key in the recent development of personalized medicine, which is becoming an essential component of modern healthcare.^8^ Coupled with early diagnostic tools, it allows individualized treatments and interventions, leading to better clinical outcomes, improved quality of life, shorter hospitalization time, and lower healthcare costs.^9^ Most chronic diseases are asymptomatic; therefore, they have often been diagnosed at their late stages. At present, only a few numbers of screening tests are available, and reliable methods for consistent biomarker sets are still limited.^10,11^

To this goal, NPs offer a valuable tool for biomarker discovery. They possess chemical structures with unique properties that differ from those of bulk materials, mostly derived from the large surface area to volume ratio.^12^ The interactions of the plasma biomolecules with the NP surface lead to the formation of layers of organic molecules onto its surface, commonly known as the biomolecular corona.^13^ This new coverage of biomolecules with high binding affinity provides the NP with different extrinsic physicochemical properties, which in turn completely change the NP’s biological identity.^14,15^ The biomolecular corona composition depends on many factors and can, in turn, influence the uptake and distribution of NPs in the body, as well as their interactions with cells and tissues.^15–21^

During the past few years, research has aimed to use the biomolecular corona not only to design and develop NP systems for biomedical applications^22^ but also to enrich low-abundance biomolecules that can trace diseases.^23^ The discovery that biological fluids such as plasma, containing diverse proteomes, may become altered during health and disease conditions founded the concept of disease-specific or *personalized* biomolecular coronas,^24^ in which NPs have a fundamental role as a potential biomarker-enriching tool.^25–27^ Recent studies have further emphasized that the composition of the biomolecular corona can vary significantly between individuals, depending on genetic background, health conditions, and even lifestyle.^28^ In fact, these person-specific biomolecular coronas have shown to modulate NP interactions with immune cells in human blood,^29^ as exemplified by influencing NP targeting of tumor cells while minimizing off-target uptake by granulocytes and monocytes in leukemia patients.^30^ Therefore, the next generation of biomarker detection techniques will be those sensitive enough to identify diseases even in the early stages of their development.^25,31,32^ For example, Corbo *et al*. used a multiprotein corona platform using a different range of NP types for early detection of Alzheimer’s disease.^26^ Proteomics analysis proved to identify Alzheimer’s disease with high specificity and sensitivity, highlighting the NP corona as a potential noninvasive diagnostic tool for early diagnosis.^26^ Recently, Trinh *et al.* exposed silica NPs to plasma from healthy and lung cancer patients.^27^ They were able to enrich the coronas with plasma fibrinogen and use them as “fingerprints” to associate the changes derived from the N-glycan peak analysis of lung cancer patients from healthy controls.^27^ These changes could be traced thanks to the abundance of proteins in the biocorona, many of which are glycosylated. As the glycosylation changes occur at early stages of a disease, their detection is believed to be an early warning biomarker for a wide range of acute and chronic diseases.^33–35^

In this study, we evaluated whether multiple NP exposures to the same blood plasma could lead to a progressive depletion of biomolecules, which would result in the isolation of a set of NP-corona complexes that carry different biomolecules for biomarker discovery applications. Previous studies have shown that the corona composition can be modulated by changing the ratio between human plasma protein mass and the NP surface area during the exposure steps, highlighting how the biological fluid plays a fundamental role in the competition of the strongly bound corona proteins.^36^ We report in this article how after numerous cycles of NP exposure and isolation from the human plasma different types of biomolecules are progressively removed, and the corona becomes enriched with low abundance biomolecules. This process can be crucial for enhancing biomarker signals, becoming a useful tool for early warning of disease detection or patient stratification.

## Experimental Details

The methods are summarized in this section. Complete details and materials can be found in the Supporting Information.

### Nanoparticle Characterization

Dynamic light scattering (DLS) measurements were performed using a Zetasizer Nano ZS (Malvern). The sample cuvettes were equilibrated at 25 °C for 90 s. Each measure’s number of runs and duration were automatically determined and repeated three times. Differential centrifugal sedimentation (DCS) experiments were performed with a CPS disc centrifuge DC24000, using the standard sucrose gradient 8–24% (Analytik Ltd.). A 544 nm PVC calibration standard was used for each sample measurement. DCS correlates the time taken for spherical particles with homogeneous density to travel from the center of the disk to the detector with their size. We assume the density of silica NPs to be 2.0 g·cm^–3^, as indicated by the manufacturer, and the protein layer density 1.125 g·cm^–3^, being an intermediate density value between the density of hydrated protein crystals and the density of blood plasma.^4^ Then, the shell thickness can be calculated using a core–shell model.^37^ Transmission electron microscopy (TEM) images were obtained with a JEOL JEM-1400PLUS transmission electron microscope operating at a voltage of 120 kV. The statistical analysis was carried out using ImageJ, where the average size and standard deviation were measured in several images containing more than 100 NPs.

### Multiple Exposure Procedure

The multiple exposure of plasma to NPs reported in this article is represented in Figure 1 and is described as follows:

**Figure 1:**
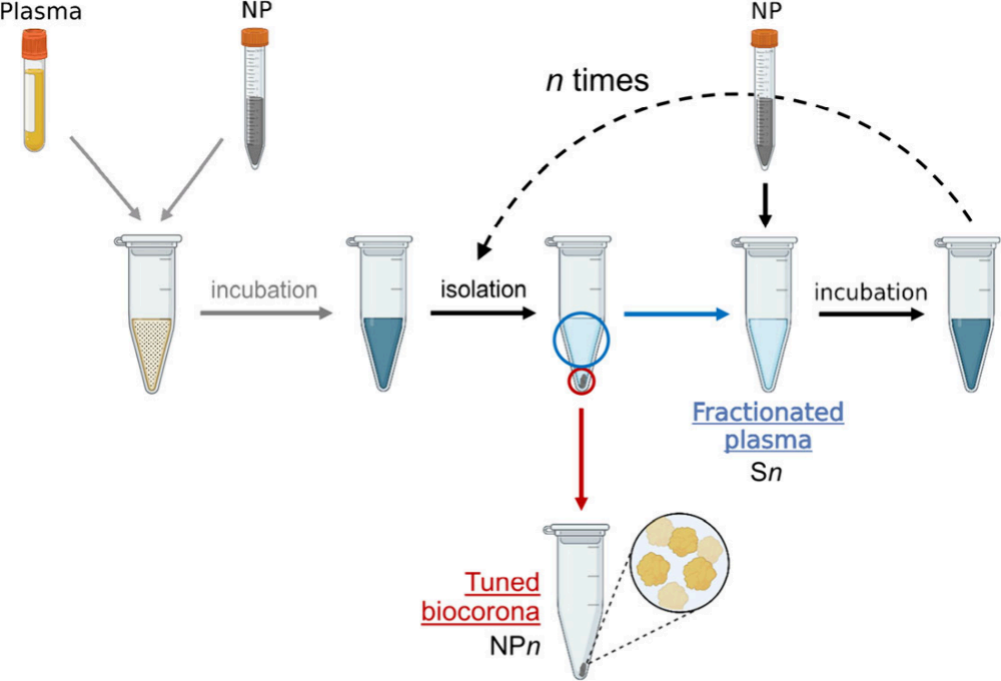
Schematic representation of the multiple exposure method to obtain tuned biocoronas and fractionated fluids. Plasma is incubated with NPs to form biomolecular coronas. Centrifugation separates NPs with a corona from the supernatant. Repeated incubation with fresh NPs produces coronas with distinct composition, yielding a “fractionated” plasma and “tuned” coronas. Created with Biorender.com.

#### 1 Fluid Preparation

Pooled plasma obtained from healthy volunteers was stored at −20 °C. On the day of the experiment, plasma aliquots were defrosted and centrifuged at 16,000 RCF for 3 min at 4 °C. The plasma sample without aggregates was pipetted, transferred to a new tube, and then diluted 10 times with PBS to a final volume of 500 μL in order to obtain 10% plasma concentration.^38^

#### 2 Exposure

The fluid is exposed to NPs and incubated for a certain time until the corona is stable. In this study, 0.5 mg of NPs was introduced at each exposure cycle, reaching a NP concentration of 1 mg/mL, and the incubation time was set to 10 min ^39^ under continuous agitation.

#### 3 Separation

Following incubation, the mixture is centrifuged (10 min, 18,000 RCF, 4 °C), obtaining the following:

- *Fractionated fluid*: The supernatant has a lower concentration of those high-affinity biomolecules that have been absorbed onto the NP surface.
- *Nanoparticle biocorona*: The pellet consists of NPs with the corona of bound biomolecules. The composition of this corona depends on how many times the fluid has been exposed to the NPs, resulting in a “tuned biocorona”. While the first coronas will be comprised of high-affinity proteins, the following batches will have different biomolecules, likely of lower affinity. This is the targeted result of the protocol, as the coronas can then be analyzed.

#### 4 Repetition of the Process

The sequence of exposure and centrifugation (points 2 and 3) can be repeated multiple times, exposing the supernatant from previous centrifugations to fresh NPs each time. The pellets can be temporarily stored in the refrigerator at 4 °C, while the supernatant plasma is transferred to another microtube and exposed to pristine NPs. In our study, this process was repeated eight more times after the initial exposure. Then, the pellets were resuspended in 500 μL of PBS, transferred to new microtubes, and centrifuged again to pellet the biomolecular corona and remove any trace of plasma. This procedure excludes biomolecular background while maintains a high fingerprint signal.^27,40^ This iteration refines the fractionation process of plasma and alters the corona composition of the NPs.

### BCA and Micro BCA

Bicinchoninic acid (BCA) assay and Micro BCA assay were conducted to measure the amount of protein in the plasma and NP corona, respectively. Protein concentrations were quantified using the BCA and Micro BCA protein assay kits, following the manufacturer’s instructions for microplate procedure, as detailed in the Supporting Information.

### SDS–PAGE

To perform the SDS–PAGE, the pellets were redispersed in 12 μL of PBS immediately after the last centrifugation step and then treated as previously reported^38^ and detailed in supplementary experimental details of the Supporting Information.

### Protein Mass Spectrometry (MS)

To determine the protein composition of the corona complexes, samples were run by SDS–PAGE, and gel bands were cut out following the in-gel trypsin digestion protocol as previously described.^38^ The samples were analyzed on a Bruker TimsTOF Pro mass spectrometer connected to an Evosep One chromatography system. Bruker mass spectrometric data from the TimsTOF were processed using the MaxQuant (version 2.0.3.0) incorporating the Andromeda search engine^41,42^ Complete details are described in the Supporting Information.

### Glycan Profiling by Liquid Chromatography (LC) and Mass Spectrometry (MS)

The *N*-glycans were released from the biomolecular corona using a LudgerZyme PNGaseFkit. For LC-ESI-MS and MS/MS analysis, procainamide labeled samples and system suitability standards were analyzed by HILIC-(U)­HPLC-ESI-MS with fluorescence detection. Complete details are located in the Supporting Information.

## Results and Discussion

In this study, commercially available silicon dioxide NPs of 100 nm in size were used due to their high colloidal stability, narrow size distribution, and wide use in biological applications.^43^ The NP size was measured by TEM, DLS and DCS to measure their nominal size and colloidal stability in solution (Table 1). Image processing from the TEM image (Figure 2a) confirmed that the NP nominal size of 100 nm. DLS analysis confirmed their high colloidal stability, with a hydrodynamic radius of 103.9 nm and a polydispersity index (PdI) of 0.03. Consistently, DCS measurements revealed a narrow size distribution peak at 95.5 nm and the absence of aggregates (Table 1).

**Table 1:** Characterization of the Pristine NP Size by Different Techniques (Value ± Standard Deviation)

TEM ± SD (nm)	DLS ± SD (nm)	DCS ± SD (nm)
95.4 ± 7.8	103.9 ± 0.7	95.5 ± 0.4

**Figure 2:**
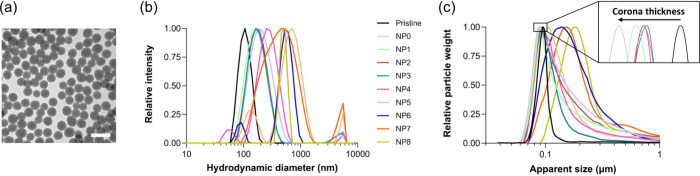
Physicochemical characterization of the pristine NPs and the NP-corona complexes. (a) TEM image of the silica NPs. Scale bar corresponds to 200 nm. (b) Hydrodynamic diameter from DLS of pristine and NPs with corona. (c) Apparent diameter from DCS of pristine and corona NPs. The formation of a corona is reflected in a shift to the left due to the change of density from the layer of biomolecules formed on the NP surface (inset).

The NPs were then exposed to human plasma, obtaining the coronas by following established protocols. While in common protocols the biological fluid is discarded after isolation of the NP-corona complexes, following NP exposure to the fluid and incubation, in this study, we exposed the pristine NPs multiple times to the same media and evaluated whether the progressive depletion of the strongly bound proteins to the NPs surface during an incubation step would change the composition of the incubated blood plasma and could lead to the isolation of a new set of biomolecules. In this study, we carried out nine cycles of NP exposure to the same blood plasma aliquot and for each cycle, the NP-corona complexes were physicochemically characterized and their corona was studied with omics techniques.

When performing the multiple exposure protocol, we termed the first set of NP-corona complexes NP0, while the supernatant was termed S0. We repeated this multiple exposure eight times, and we isolated NP1–NP8, which are the NP-corona complexes, and then S8, which corresponds to the blood plasma after the nine exposures.

The NP-corona complexes were characterized by DLS and DCS (Figure 2b,c). The analysis from DLS showed an increase in the hydrodynamic size compared with the pristine NPs as a result of the biomolecular corona formation. With the increase of the exposure, no changes in the hydrodynamic size were observed until the NP3 sample, indicating that from NP0 to NP3, the potential removal of high-affinity biomolecules from the media had little effect on the NP colloidal stability (Table 2). However, sample NP4 increased the hydrodynamic size and the polydispersity, and from samples NP5 to NP8 the colloidal stability is decreased. DCS analysis showed a first shift toward a smaller apparent size compared to the pristine material, which is expected for NPs that have a higher density than the coating biomolecules, where the protein deposition leads to a reduced NP-corona density and, therefore, a delay in sedimentation.^4,38^ The sequential exposure of plasma to NPs led to a progressive peak shift to the right. A core–shell model was applied to correlate the NP-corona peak with the pristine NP and obtain quantitative information on the corona layer thickness.^37^ This analysis revealed a progressive decrease in shell thickness from NP0 to NP2, indicating that multiple additions of NPs to the plasma lead to progressively smaller protein coronas (Table 2). The shell thickness values remained similar for samples NP2 to NP4, but for samples NP5 to NP8 these values could not be calculated as the peak was shifted to larger apparent sizes than the pristine material due to agglomeration and/or aggregation (as indicated by DLS, where an increase of the PdI is observed). MicroBCA analysis was also carried out to measure the protein amount in the NP-corona complexes after each exposure.^44^ Interestingly, we observe a trend where the amount of protein adsorbed to the NPs decreases with successive exposures of NPs to the fluid, which is in line with other results. Overall, the physicochemical characterization by DCS and protein quantification by microBCA and SDS–PAGE show a consistent decrease in corona content upon repeated exposure. However, the magnitude of this decrease differs between methods, likely due to limitations in both. The BCA assay is influenced by protein composition and assumes uniform response across proteins,^45^ while the DCS core–shell model assumes constant corona density and geometry, and its thickness cannot be directly correlated with protein amount. Consequently, protein mass and shell thickness do not scale linearly, despite both trends showing directional agreement.

**Table 2:** Hydrodynamic (DLS) and Apparent Sizes (DCS) of the NP Samples as Well as the Calculated Corona Thickness and the Protein Content^a^

	DLS ± SD, *Z*-average (nm)	DLS ± SD, PdI	DCS ± SD, main peak (nm)	DCS ± SD, corona thickness (nm)	protein amount in the corona (μg_protein_/mg_NP_) ± SD
pristine	103.9 ± 0.7	0.03 ± 0.01	95.5 ± 0.4	0	0
NP0	169.9 ± 10.8	0.17 ± 0.06	88.6 ± 0.8	13.5 ± 2.4	136.9 ± 14.8
NP1	173.6 ± 10.0	0.20 ± 0.04	90.4 ± 0.9	8.9 ± 2.0	126.5 ± 11.9
NP2	164.6 ± 3.6	0.16 ± 0.01	91.4 ± 0.3	6.7 ± 0.6	124.0 ± 11.1
NP3	175.3 ± 7.9	0.19 ± 0.04	91.3 ± 1.1	7.1 ± 2.2	122.1 ± 10.0
NP4	215 ± 33.3	0.26 ± 0.06	91.2 ± 1.0	7.1 ± 2.0	125.2 ± 12.8
NP5	468.8 ± 202.0	0.48 ± 0.16	105.5 ± 2.7		123.9 ± 17.2
NP6	547.6 ± 45.5	0.52 ± 0.17	138.2 ± 8.9		98.2 ± 18.3
NP7	443.8 ± 79.7	0.41 ± 0.14	154.6 ± 12.1		102.4 ± 12.5
NP8	621.6 ± 205.3	0.56 ± 0.38	184.0 ± 17.3		97.8 ± 13.5

a Mean and standard deviation were calculated over three technical replicates.

Proteomics and glycan analyses were carried out in samples NP0, NP1, NP2, NP4, NP6, and NP8 to identify changes in biomolecular composition of both the corona and supernatant between the different exposure cycles. These samples were selected as the most representative subset because the corona patterns exhibited greater changes in SDS–PAGE.

Figure 3a shows an SDS–PAGE gel of the NP-protein composition where a progressive alteration in band intensity and distribution from NP0 to NP8 occurs, indicating a change in the protein corona composition as a result of multiple exposures to the plasma sample. In particular, NP0, NP1, and NP2 display similar band patterns with four dominant bands at molecular weights of around 45–70 kDa. From the NP3 exposure, the intensity of the doublet bands between 49 and 56 kDa, later identified as β and γ chains of fibrinogen by MS, decreases significantly (Figure 3a,b).^46^ However, a band of 52 kDa appears from N4 to N8, suggesting a fibrinogen displacement. Gel bands of 175 and 37 kDa increase within intensity from NP0 to NP3, but the expression decreases from NP4 onward, suggesting a transient enrichment. A second band appears above the band at 30 kDa, corresponding to apolipoprotein A1,^27,47^ as plasma is exposed repeatedly to the NPs. Other differences across the gel bands were observed at different molecular weights during multiple exposure cycles. Figure 3b shows the gel band densitometry, highlighting the protein pattern changes across the samples. Overall, these findings suggest that progressive exposure of blood plasma to NPs leads to a depletion of selective proteins that have an affinity toward the particle’s surface. Moreover, the depletion is not directly correlated to the original protein abundance in the fluid, as the relative abundance of the gel band at 66 kDa, which corresponds to serum albumin, remains constant (Figure 3b). However, the increase in exposure leads to a change in the protein amount of certain biomolecules in blood plasma, correlated to an enrichment of different biomolecules on the NP surface. The same experiment was also repeated with washed corona samples, where we observed a similar depletion profile (Figure S1). The sample control with 10% blood plasma only was also processed with the NP0 preparation, and low protein background was detected (Figure S2, Figure S3) where the background is negligible when compared to the NP-corona signal. We also evaluated whether a similar depletion observed in Figure 3a could be achieved by exposing the same amount of NPs used for the eight multiple exposures (4 mg in total) in one single incubation. Despite the fluid was fractionated, this approach did not result in a distinct prefractionation of glycoproteins as shown in Figure 3, indicating that progressive glycoprotein removal, driven by affinity to the NP surface, is necessary for selective fractionation (Figure S4). In particular, the progressive NP exposure to blood plasma leads to the removal of high affinity proteins. However, with the increase in the exposure, high affinity proteins are depleted, leading to the adsorption to proteins with progressively lower affinity.

**Figure 3:**
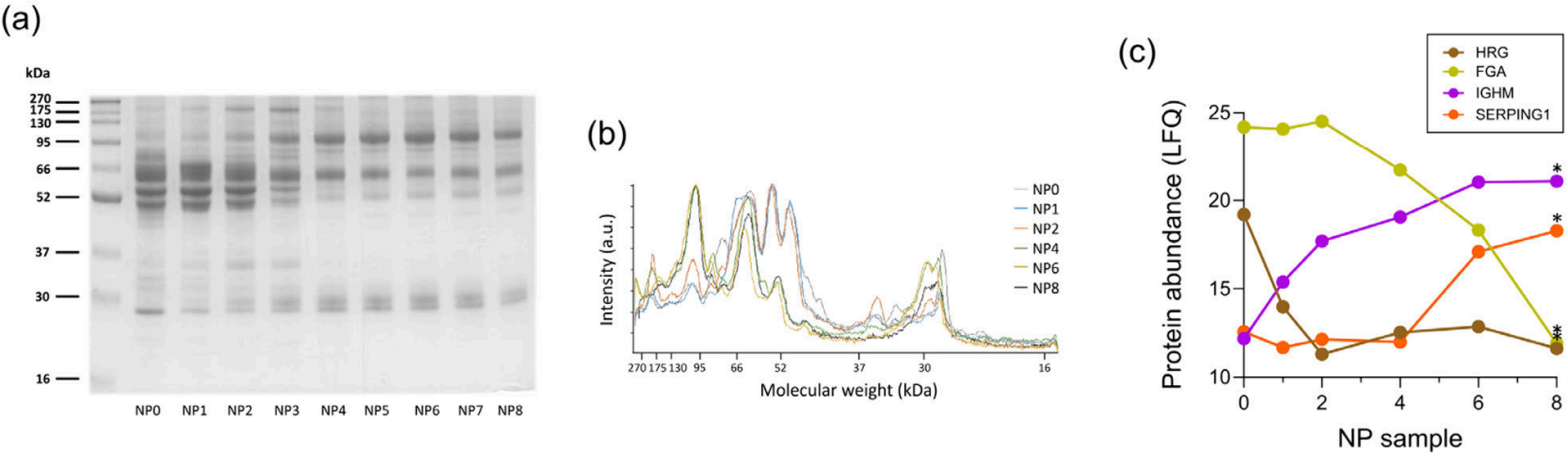
Comparative analysis of the protein profiles from the NP corona samples. (a) SDS–PAGE and (b) corresponding densitometry analysis for NP0–NP8. (c) Example of proteins identified in the corona by mass spectrometry (Table S1) changing their abundance in the NP corona through the different samples of the multiple exposure method. (*t* test, *p < 0.1).

MS analysis was conducted to identify the proteins present in each corona and assess their relative abundance across the samples using label-free quantification (LFQ). The data revealed that certain proteins, such as fibrinogen, apolipoprotein A1, and apolipoprotein E, which were highly abundant in early samples, decreased in later samples (Figure 3c, Table 3). Histidine-rich glycoprotein (HRG) followed a similar trend, showing a sharper decline as the exposure progressed (Figure 3c, Table S1). In contrast, immunoglobulins like IgM (IGHM), complement proteins and inhibitors, such as SerpinG1 (SERPING1, a glycoprotein recently reported as a biomarker for poor collateralization in patients with stable angina and chronic total occlusion^48^), increased in abundance in the later samples, indicating a shift in the protein composition (Figure 3c).

**Table 3:** Top 10 Proteins Identified in the NP Corona and the Values of Their Label-Free Quantification (LFQ) in NP0 and NP8^a^

NP0	Uniprot	MW	jog_2_(LFQ) ± SD	NP8	Uniprot	MW	log_2_(LFQ) ± SD
Fibrinogen (α, β, γ chain)	P02671, P02675, P02679	95.0, 55.9, 51.5	24.2 ± 0.2, 21.9 ± 0.7, 22.3 ± 0.7	Albumin	P02768	69.4	23.7 ± 2.0
Albumin	P02768	69.4	23.3 ± 2.8	Ig κ chain C	P01834	11.8	22.1 ± 0.6
Ig γ3 chain C	P01860	49.1	20.2 ± 2.7	Ig γ3 chain C	P01860	49.1	21.8 ± 0.9
Histidine-rich glycoprotein	P04196	59.6	19.4 ± 1.1	Complement C3	P01024	187.2	21.3 ± 1.2
Apo E	P02649	36.2	19.3 ± 0.8	Ig α1 chain C	P01876	42.8	21.2 ± 1.2
Coagulation factor XII	P00748	67.8	19.2 ± 0.4	Ig μ chain C	P01871	51.9	21.1 ± 1.4
Apo A-I	P02647	30.8	19.0 ± 2.0	Kininogen-1	P01042	72.0	20.2 ± 1.9
Complement C4	P0C0L4	192.8	18.6 ± 2.3	Ig γ1 chain C	P01857	43.9	20.1 ± 0.5
Ig α1 chain C	P01876	42.8	17.8 ± 2.5	Inter-α-tryp inhibitor H2	P19823	106.5	19.9 ± 2.4
Kininogen-1	P01042	72.0	17.6 ± 1.2	Complement factor B	P00751	85.5	19.7 ± 2.3

a Protein names are abbreviated. Complete details are found in Table S1.

Similarly, kininogen, a glycoprotein that modulates the intrinsic pathway of coagulation and whose glycosylation changes have been associated with the early detection of hepatocellular carcinoma,^49^ as well as serum amyloid P-component, a protein of the innate system whose reactivity is modulated by glycosylation,^50^ become more abundant over the exposure cycles (Table S1). This shift toward a more immunoglobulin-rich corona highlights the multiple exposure potential to enrich selectively certain protein types, which could have significant implications for disease diagnosis.

HILIC chromatography was then carried out to evaluate *N*-glycan changes across the samples. The *N*-glycan analysis aimed to trace changes in the adsorbed glycoconjugates and correlate them with potential biomarkers. Figure 4a shows the chromatograms with the retention time and glycan peaks (GP), and Table S2 contains the identified glycans for each peak with MS. Specific glycan structures are annotated with symbols representing different monosaccharides. In the NP corona glycan profile, there is a notable variation in the intensity and distribution of some peaks across samples of multiple exposures, from NP0 to NP8, that reflects the changes in the protein compositions in the corona. For instance, the peak of A2G2S1 glycan is enriched in the corona samples compared with the untreated blood plasma (Figure 5c). However, the GP16 intensity decreases with the increase of exposure, suggesting a decrease in the enrichment of fibrinogen in the corona (Figure 4b). This finding is in agreement with the proteomics analysis where fibrinogen is a top plasma corona protein, and the concentration decreases with the increase of the NPs’ exposure to human plasma (Figure 3a,c). After exposing plasma multiple times to NPs, the glycan peaks that become abundant in the corona are related to the most abundant in human IgG, which is typically biantennary with terminal *N*-acetylglucosamine (GlcNAc), monogalactosylated, digalactosylated, one or two sialic acids, and each of these with or without core fucose and bisecting GlcNAc.^51^

**Figure 4:**
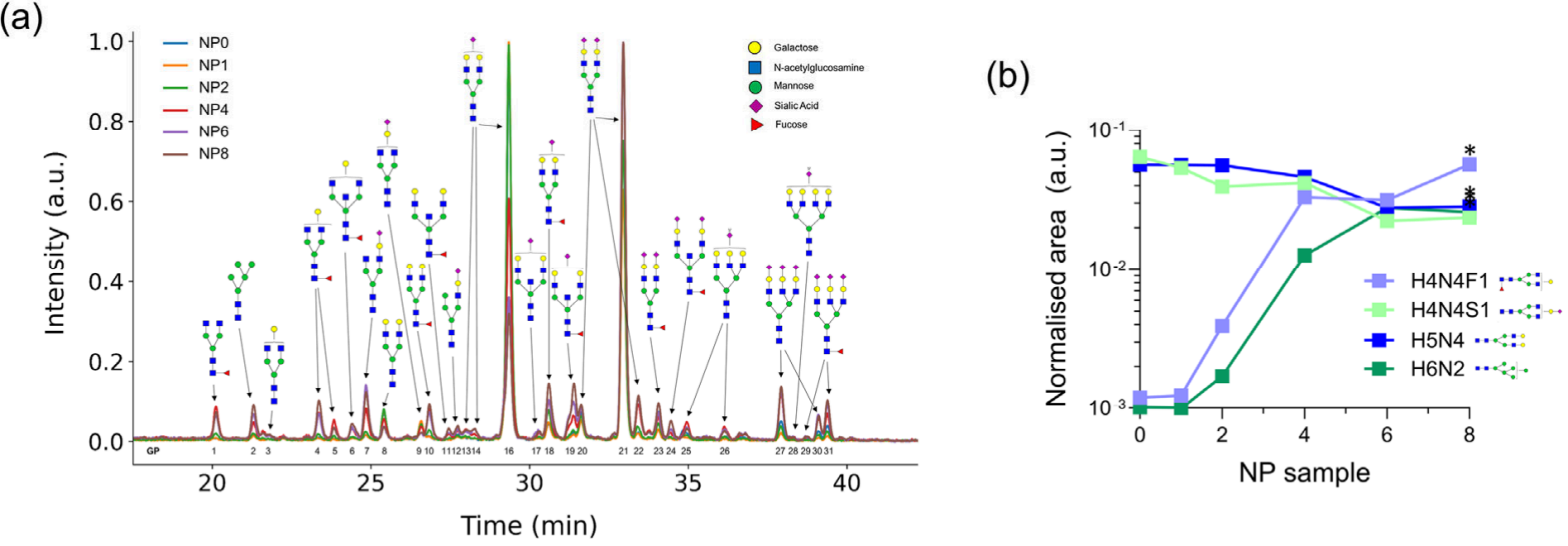
Comparative analysis of the glycan profiles from the NP corona samples. (a) UHPLC profiles of the glycan moieties embedded in the corona. (b) Example of glycan compositions changing their abundance in the NP corona over the different samples of the multiple exposure method. The glycan cartoons shown are for illustrative purposes only; precise features like isomer type would have to be confirmed by other analytical means (*t* test, *p < 0.05).

**Figure 5:**
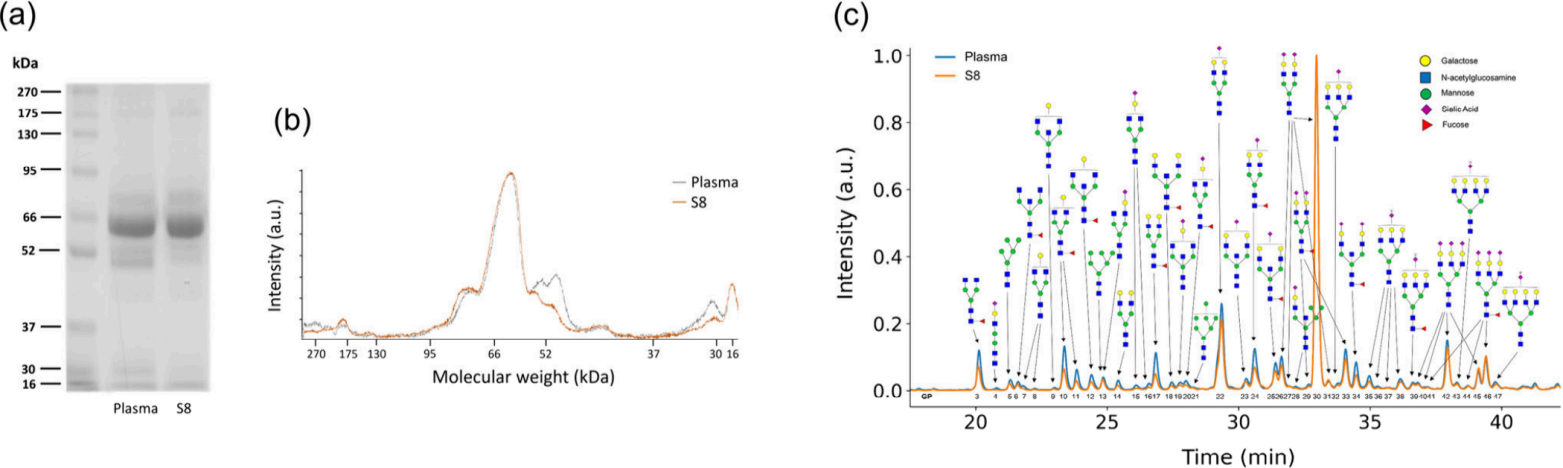
Comparative analysis of protein and glycan profiles from plasma samples. (a) SDS–PAGE and (b) densitometry for the plasma and S8 after multiple exposure to NPs. (c) Chromatograms corresponding to the glycan profiles of plasma and S8. The glycan cartoons shown are for illustrative purposes only; precise features like isomer type would have to be confirmed by other analytical means.

Glycan traits were later analyzed by using glycan MS data. The main observation from the relative areas of each glycan peak across samples is that many different glycan structures increased sequentially (Table 4, Table S3). In fact, less than 10% of the identified glycans showed a decrease in abundance in the corona from NP0 to NP8, while the majority significantly increased (Figure 4b, Table 5). This glycan composition confirms an enrichment process from human plasma, with profiles becoming more diverse as exposures increase.

**Table 4:** Top 10 Glycans Identified in the NP Corona^a^

NP0	NP1	NP2	NP4	NP6	NP8
A2G2S1	A2G2S1	A2G2S1	A2G2S2	A2G2S2	A2G2S2
A2G2S2	A2G2S2	A2G2S2	A2G2S1	A2G2S1	A2G2S1
A2G1S1	A2G2	A2G2	A3G3S3	FA2BG2S1	FA2BG2S1
A2G2	A2G1S1	A2G1S1	FA2G2S1	A3G3S3	FA2G2S1
FA2G2S2	FA2G2S1	FA2G2S1	FA2G2	FA2G2S1	A3G3S3
FA2G2S1	FA2G2S2	FA2G2S2	A2G2	FA2G2	FA2G2
A3G3S3	A3G3S3	A3G3S3	A2G1S1	FA2G2S2	FA2G1
A1G1S1	A1G1S1	FA2G2	FA2G2	FA2G1	FA2G2S2
M4A1G1S1	FA2BG2S1	FA2BG2S1	FA2BG2S1	FA2G2	A3F1G3S3
A3F1G3S3	FA2G2	M9	FA2G1	A2G2	FA2BG1

a The correspondence of these notations to the glycan composition is found in Table S4.

**Table 5:** Top 10 Glycans with a Higher Increase and Higher Decrease Identified in the NP Corona^a^

glycan composition	suggested glycan oxford notation	mass (m/z)	retention time (min)	ratio (NP8/NP0) ± SD
H4N4F1	FA2G1	922.9	23.2	47.4 ± 1.0
H4N5F1	FA2BG1	1024.4	24.7	32.8 ± 1.3
H5N5F1S1	FA2BG2S1	1251.0	30.6	29.2 ± 3.8
H8N2	M8	970.9	30.5	27.3 ± 3.0
H3N4F1	FA2	841.9	20.0	27.1 ± 0.8
H4N5F1	FA2BG1	2047.9	24.3	27.1 ± 1.1
H3N5F1	FA2B	943.4	21.5	26.6 ± 1.3
H6N2	M6	1616.7	24.4	24.9 ± 1.0
H5N5F1	FA2BG2	2209.9	27.4	23.8 ± 1.4
H4N3F1	FA1G1	821.4	21.3	21.5 ± 1.9
H5N4F1S1	FA2G2S1	1149.5	30.5	0.99 ± 0.17
H3N3S1	A1G1S1	812.8	20.7	0.97 ± 0.48
H7N6F1S3	A4F1G4S3	1204.1	41.6	0.84 ± 0.29
H6N4S1	M5BA1G1S1	2313.9	32.0	0.79 ± 0.04
H3N3S1	A1G1S1	812.8	20.7	0.60 ± 0.30
H5N4S1	A2G2S1	1076.4	29.2	0.51 ± 0.09
H5N4	A2G2	1860.8	25.3	0.50 ± 0.03
H4N5S1	A2BG1S1	1096.9	27.7	0.49 ± 0.02
H4N4S1	A2G1S1	995.4	26.4	0.37 ± 0.02
H8N7S4	A4G4S4	2060.8	44.5	0.24 ± 0.11

a The correspondence of these notations to the MS values and glycan composition is found in Table S3 and Table S4, respectively.

For instance, specific glycan compositions (and derived structures) like H4N4F1 (or FA2G1), H4N5F1 (or FA2BG1), H5N5F1S1 (or FA2B2S1), and H3N5F1 (or FA2) are abundant glycan structures found in immunoglobulins. These glycans show significant enrichment over time, with fold changes from NP0 to NP8 ranging from 47 to 27, as indicated in Table 5.

There is a noticeable change in the monosaccharide composition between the different samples. In fact, Man7 and Man8, which are mostly abundant in IgG, were both detected in NP0 and NP8, but the amount of oligomannose glycans increased as we progressed with the exposure, indicating a different enrichment of glycoproteins.

Mannose structures are considered biomarkers for signature for coronary artery disease, where glycan changes were associated with coronary angiographies, and it is a potential noninvasive predictor of adverse outcomes.^52^ Moreover, mannose structures are also found in HIV-1 envelope glycoprotein, which plays a key role in viral immune evasion by forming a “glycan shield”.^53^ The multiple exposures enriched the presence of fucosylated glycans, and structures such as FA2G1 and FA2BG1 have been connected to Crohn’s disease.^54^ The multiple exposures also resulted in the enrichment of bisected GlcNac glycan species, where glycan structures like FA2BG2 and FA2BG2S1 have been associated with Alzheimer’s disease.^55^ In turn, terminal galactose and sialic acid peaks showed a variable presence across all samples. The presence of sialylated structures and changes in these associated glycan structures have been associated with some diseases such as diabetes, cardiovascular disease (CVD), and other chronic diseases.^56^ In particular, these changes in protein glycosylation, such as IgG, are related to the stimulation of immunological and inflammation pathways, as evidenced in inflammatory disorders with rheumatoid arthritis being a notable example.^57^ Additionally, the level of IgG galactosylation (hence the abundance of the FA2 glycan structures) has been associated with response to methotrexate in arthritis.^58^ Changes in FA2 glycans and in galactose are also associated with natural aging but also with inflammation; therefore these glycans could be used as a biochemical age biomarkers.^59^

Specific glycans have also been linked during the last decades to particular cancers, serving as potential biomarkers. In particular, A2G2S1 is associated with both prostate cancer^60^ and stage III ovarian cancer,^61^ while changes in A2G2S2, A2BG2S2, A4G4S3, A4G4S4, FA1, FA2, FA2B, and M4A1G1S1 are specifically linked to ovarian cancer.^62^ Additionally, FA2BG1S1 has been associated with both ovarian cancer^62^ and colorectal cancer,^63^ while other glycans like FA2G2S1, FA2BG2, A2, and FA2G2 are just linked to colorectal cancer.^63^ Other structures such as A3G3S3 and FA2G2S2 have been recently identified as potential biomarkers for lung cancer.^27^ In fact, altered glycans in liver cancer have been linked to affecting key proteins that drive tumor progression.^64,65^

The identification and enrichment of these glycans on NPs could enhance diagnostic precision, allowing for earlier and better detection of diseases. Therefore, this multiple exposure method allows selective enrichment within the plasma glycome, also leading to a personalized design of coronas in terms of not only proteins but also glycans, which in turn could significantly impact the biological identity and the subsequent physiological interactions of these NPs.

The changes in the multiply exposed fluid were also accounted for. Figure 5a shows the SDS–PAGE of the full plasma (untreated) and the plasma supernatant after the multiple exposure treatment (S8). Despite the main band corresponding to albumin, no significant changes can be detected, with the exception of two gel bands of around 50 kDa and a band of 30 kDa, which are decreased in the S8 compared to the plasma control (Figure 5b). In the chromatogram profile from the glycan analysis (Figure 5c), a rich set of peaks is shown across the retention time, indicating a complex glycan composition and reflecting a high glycan abundance in the plasma. Interestingly, there is a significant difference between the untreated plasma and the corona profile (Figure 4a), indicating the capacity of the corona to enrich specific glycan features, as demonstrated in previous studies.^27,66^ When the plasma profile is compared to S8, we notice that the main difference is the peak intensity. The intensity of most corona-derived glycan structures appears to be reduced in S8, which implies that NPs have fractionated the plasma by adsorbing glycoconjugates, thereby reducing their presence in the fluid. Therefore, glycoprotein depletion from the plasma can be directly correlated with selective enrichment by the NPs. These findings highlight that plasma’s biological functions after NP exposure could be affected, potentially altering immune recognition and other glycan-mediated processes.

## Conclusions

In this work, we introduce the multiple exposure method, a process by which NPs selectively adsorb biomolecules and, at the same time, fractionate plasma. As we expose pristine NPs to plasma sequentially, we observe a pronounced transition of the high-affinity glycoproteins forming the corona, which results in a change in protein and glycan signal. These changes are evidenced by the preferential binding of proteins such as fibrinogen to the NPs surface, and as they are sequentially removed from the plasma, different protein and glycan structures emerge in the corona.

These findings show the capacity of NPs to act as a sorting mechanism within biological samples, sequestering specific proteins and glycans and allowing for the exposure of less abundant, potentially more biologically relevant species. We believe that the results from this study will set a novel mechanism in which one can get personalized coronas by exposing the same fluid over and over to pristine NPs, shifting from a corona of high-affinity biomolecules to a corona of lower-affinity biomolecules. Moreover, by employing different nanomaterials, concentrations, sizes, or surface chemistry, one would be able to obtain different coronas and different fluid lavages. We hope that the translation of this multiple exposure protocol can help the designing of NP corona of interest and particularly lead to a new generation of biomarking devices, becoming a useful proteomics and glycomics tool for the early diagnosis and monitoring of diseases.

## Supplementary Material



## Data Availability

The data underlying this study are openly available in Zenodo at https://doi.org/10.5281/zenodo.15050700.

## Notes

Supplementary experimental details on materials and methods (Figures S1–S4), and supplementary data (Tables S1–S4) (PDF)
