# Supplementary material for: Multiple Exposures of Plasma to Nanoparticles: A Novel Tool to Personalize Biomolecular Coronas and Fractionate Fluids
Source: Anal Chem. 2025 Jun 30;97(27):14132–41. doi: 10.1021/acs.analchem.4c05573 (PMC12268827; doi:10.1021/acs.analchem.4c05573)
Supplement: Supplementary file 1 [file ac4c05573_si_002.pdf]

SUPPORTING INFORMATION

Multiple exposures of plasma to nanoparticles: a novel tool to personalize biomolecular coronas and fractionate fluids

Alberto Martinez-Serra,[a] Jack Cheeseman,[b] Asia Saorin,[a] Mahmoud G. Soliman,[a] Marko Dobricic,[a] Daniel I. R. Spencer,[b] and Marco P. Monopoli*[a]

(a) Chemistry Department, Royal College of Surgeons in Ireland (RCSI), 123 St Stephen's Green, Dublin 2, Ireland.

(b) Ludger Ltd., Culham Campus, Abingdon, OX14 3EB, Oxfordshire, United Kingdom

ABSTRACT: Nanoparticles (NPs) have emerged as a valuable tool for biomarker discovery due to their ability to interact with biological fluids and form biomolecular coronas. In this study, we introduce a multiple exposure method that uses NPs to fractionate biological fluids and obtain personalized coronas. By repeatedly exposing plasma to silica NPs, we observed a progressive change of biomolecule profile in both pellet and supernatant. The varying protein and glycan composition of the corona was characterized using techniques such as SDS-PAGE, mass spectrometry, and UHPLC. Notably, the corona's composition evolved with each exposure cycle, reflecting the selective binding of proteins and glycosylated molecules from a corona of high-affinity biomolecules to a more diverse corona with very distinct structures. By tracing the sequential modification of protein and glycan composition, we believe that the method can be useful to trace specific biomarker profiles, offering a non-invasive alternative to conventional diagnostic processes with the potential to become a useful tool for disease monitoring and advanced biomedical applications.

1. SUPPLEMENTARY EXPERIMENTAL DETAILS

1.1. Materials

Silica NPs of 0.1µm (stock concentration of 50 mg·mL-1) were provided by Kisker Biotech GmbH (Germany). Phos-phate buffer saline (PBS) tablets, Eppendorf LoBind micro-centrifuge tubes, tris base (99%), glycine, acrylamide/bis-acrylamide 40% solution, sodium dodecyl sulfate (SDS, 99%), ammonium persulfate (APS), N, N, N', N'-tetramethylethylenediamine (TEMED), and D-(+)-Sucrose (99.9%) were purchased from Sigma-Aldrich (Ireland). We dissolved one PBS tablet in 200 mL of ultrapure water to obtain a 0.01 M phosphate buffer, 0.0027 M potassium chloride and 0.137 M sodium chloride solution (pH 7.4 at 25°C). The 3x Blue Loading Buffer and 30X Reducing Agent (1.25 M DTT) were purchased from Cell Signaling Technol-ogy (Ireland). The Prime-Step prestained protein ladder was purchased from BioLegend (Ireland). The BCA kit, micro BCA kit, Pierce C18 Tips and Imperial™ Protein Stain were purchased from Thermo Fisher Scientific (TFS, Ireland). TEM grids of Formvar / Silicon monoxide 200 mesh with copper approximate grid hole size 97µm were purchased from Ted Pella (USA). The LudgerZyme PNGaseF Kit, LudgerTagProcainamide Glycan Labeling Kit, and Ludger-Clean ProcainamideClean-up Plate were purchased from Ludger Ltd. (U.K.). Human plasma from eight healthy do-nors provided by the Irish Blood Transfusion Service (IBTS) was mixed in equal proportions to obtain average pooled plasma. All the plasma sources were prepared from whole blood using EDTA tubes. The total protein concentrations were measured with BCA, following the manufacturer's in-structions. Access and use of plasma samples were covered by the RCSI Ethics number 001246b.

1.2. BCA and Micro BCA

Plasma samples were diluted one hundred times, and the supernatant was diluted ten times. 200 µl of working reagent was added to 25 µl of samples in each well, followed by a 30-minute incubation at 37°C. BSA solutions were used to create a calibration curve for reference, with concentrations ranging from 2000 µg·mL-1 to 25 µg·mL-1. All plasma samples, along with the standards and a blank containing only PBS, were run in triplicates. To quantify the amount of protein in the corona, NP pellets were resuspended in 500µl of PBS after centrifugation. BSA standards with concentrations ranging from 200 µg·mL-1 to 0.5 µg·mL-1 were used for the calibration curve. 150 µl of Micro BCA working reagent were added to 150 µl of all samples in triplicates – standards, corona samples, and NP controls – and were incubated at 37 °C for 2 hours. Absorbance at 562 nm was measured using a Tecan Infinite 200 Pro microplate reader for both procedures. The BCA values were reported as µgprotein/mgNP of NPs and apply also for µgprotein·mL-1 as the total incubation volume was 0.5 mL.

1.3. SDS-PAGE

To perform the SDS-PAGE, the pellets were re-dispersed in 12µl of PBS immediately after the last centrifugation step and mixed with 3× loading buffer (62.5 mM Tris–HCl pH 6.8, 2% (w/v) SDS, 10% glycerol and 0.01% (w/v) bromophenol blue), heated at 100 °C for 5 min and loaded for running using the Prime-Step Prestained Broad Range Protein Ladder (6.5–270 kDa) as a molec-ular weight marker. 2µl of the samples were loaded in each well of 8 and 10% polyacrylamide gels prepared in the lab on the same day. Gel electrophoresis was performed with a Tris-glycine buffer on a Mini-PROTEAN electrophoresis system (Bio-Rad) at a constant voltage of 120 V, 400 mA for about 1.5h until the proteins neared the end of the gel. The gels were stained with Coomassie blue staining following the manufac-turer's guide. Gels were scanned using an Amersham Imager 600 (GE Healthcare Life Sciences). Densitometry analysis was performed using ImageJ.

1.4. Protein Mass Spectrometry (MS)

To determine the protein composition of the corona complexes, samples were run by SDS-PAGE, and gel bands were cut out following the in-gel trypsin digestion protocol as previously described.[1] Digested peptides were then resuspended in 40µl of 0.1% formic acid. Then the peptides (1000ng, quantified by Nanodrop) were loaded onto Evotips as per manufacturer's instructions (EvoSep). Briefly, Evotips were activated by soaking them in isopropanol, primed with 20 µL buffer B (ACN, 0.1% FA) by centrifugation for 1 min at 700 g. Tips were soaked in isopropanol and equilibrated with 20 µL buffer A (MS grade water, 0.1% FA) by centrifugation. Another 20 µL buffer A was

loaded onto the tips and the samples were added on top of that. Tips were centrifuged and washed with 20 μl buffer A followed by overlaying the C18 material in the tips with 100 μL buffer A and a short 20 s spin.

The samples were analysed by the Mass Spectrometry Resource (MSR) at University College Dublin on a Bruker TimsTOF Pro mass spectrometer connected to an Evosep One chromatography system. Peptides were separated on an 8 cm analytical C18 column (Evosep, 3 μm beads, 100 μm ID) using the pre-set 30 samples per day gradient on the Evosep one. The Bruker TimsTOF Pro mass spectrometer was operated in positive ion polarity with TIMS (Trapped Ion Mobility Spectrometry) and PASEF (Parallel Accumulation Serial Fragmentation) modes enabled. Data-Dependent Acquisition (DDA) was used for peptide analysis. The accumulation and ramp times for the TIMS were both set to 100 ms, with an ion mobility (1/k0) range from 0.62 to 1.46 Vs/cm. Spectra were recorded in the mass range from 100 to 1,700 m/z. The precursor (MS) Intensity Threshold was set to 2,500 and the precursor Target Intensity was set to 20,000. Each PASEF cycle consisted of one MS ramp for precursor detection followed by 10 PASEF MS/MS ramps, with a total cycle time of 1.16 s.

Bruker mass spectrometric data from the TimsTOF was processed using the MaxQuant (version 2.0.3.0) incorporating the Andromeda search engine.[2, 3] To identify peptides and proteins, MS/MS spectra were matched against a Uniprot *Homo sapiens* (Human) database containing 82,518 entries (proteome ID UP000005640). All searches were performed using the default setting of MaxQuant, with specific enzyme Trypsin/P digestion mode and a false discovery rate of 1% on the peptide and protein level. The database searches were performed with acetylation (protein N terminus) and oxidation (M) as variable modifications. For the generation of label-free quantitative (LFQ) ion intensities for protein profiles, signals of corresponding peptides in different nano-HPLC MS/MS runs were matched by MaxQuant in a maximum time window of 1 min. Perseus statistical software (version 2.0.3.0) was used to analyse the LFQ intensities.[4] The data were $\log_2$-transformed, and missing values were imputed from a normal distribution by randomly sampling from a normal distribution of 1.8 standard deviations from the mean and a width of 0.3 standard deviations, in order to approximate the distribution of low-abundance proteins.[5]

1.5. Glycan profiling by Liquid Chromatography (LC) and Mass Spectrometry (MS)

The N-glycans were released from the biomolecular corona using a LudgerZyme PNGaseFkit. Briefly, the corona was resuspended in 15μL of ultrapure water. A volume of 10μL of 10×denaturation solution was added to each sample and mixed. The samples were incubated for 10 min at 100°C. The sample tube was briefly vortexed and centrifuged at 18,000 RCF for 10 min to remove NPs. A volume of 20μL of 10×reaction buffer, 20μL of 10% NP-40 solution, 135μL of pure water, and 1μL of PNGaseF were added to each supernatant containing glycoproteins. Samples were vortexed and incubated overnight at 37°C (14−16 h). For fluorescent labeling, 200μL of each sample was transferred to a nonskirted 96-well PCR plate (300μL, 4titude Ltd.) and the samples dried down over 9 h. The released N-glycans were converted to aldoses with 40μL of 0.1% formic acid over 45 min, filtered through a 96-well protein binding plate, and dried down completely over 9 h. Released N-glycans were fluorescently labeled by reductive amination with procainamide using a LudgerTag Procainamide Glycan Labeling Kit. Briefly, samples were incubated for 60 min at 65°C with 20μL of procainamide labeling solution. The procainamide labeled N-glycans were cleaned up using a HILIC-type purification Ludger-Clean Procainamide Clean-up Plate on a vacuum manifold. The purified procainamide labeled N-glycans were eluted with pure water (300μL). For LC-ESI-MS and MS/MS analysis, procainamide labeled samples and system suitability standards were analysed by HILIC-(U)HPLC-ESI-MS with fluorescence detection. To 25μL of each sample was added 75μL of acetonitrile. A volume of 25μL of each sample was injected onto an ACQUITY UPLC BEH-Glycan 1.7μm,2.1 mm×150 mm column (Waters) at 40°C on an Thermoscientific Vanquish UHPLC instrument with a fluorescence detector (λ_{ex}= 310 nm, λ_{em}=370 nm), attached to a Thermoscientific Orbitrap Exploris 120 mass spectrometer. The chromatography conditions used were: Solvent A 50 mM ammonium formate pH 4.4 made from Ludger Stock Buffer, and solvent B acetonitrile. Gradient conditions were 0 to 53.5 min, 76 to 51% B, 0.4 mL·min^{-1}; 53.5 to 55.5 min, 51% to 0% B, 0.4 mL·min^{-1} to 0.2 mL·min^{-1}; 55.5 to 57.5 min,0% B at a flow rate of 0.2 mL·min^{-1}; 57.5 to 59.5 min, 0 to 76% B, 0.2 mL·min^{-1}; 59.5 to 65.5 min, 76% B, 0.2 mL·min^{-1}; 65.5 to 66.5 min,76% B, 0.2 mL·min^{-1} to 0.4 mL·min^{-1}; 66.5 to 70.0 min, 76% B, 0.4 mL·min^{-1}. Relative abundances of the peaks were obtained directly after normalisation using HappyTools.[6]

The Thermoscientific Orbitrap Exploris 120 mass spectrometer settings were: source temperature, 300°C; gasflow, 10 L·min^{-1}; capillary voltage, 3500 V; max accu time, 100 ms; positive ion mode; mass range scanned, 500−2500, isolation window (m/z), 2; collision energy type, normalised; HCD, 20%; resolution, 15000; intensity threshold, 2.0e5; type of experiment, ddMS2. Data integration of the MS1 spectra was performed using Skyline (version 24).[7] The mass

spectrometry data for N-glycans was analysed using Thermoscientific Freestyle software (version 1.8) and the structures were manually assigned. Assignments were made based on exact mass, and the accuracy of the actual mass compared to the theoretical mass being below 20ppm, fragmentation data and retention order. Further to this, reference was made to previously reported glycan structures found in plasma and serum.[8, 9] N-glycan compositions were illustrated to the Consortium for Functional Glycomics (CFG) notation[10]: N-acetylglucosamine (N; blue square), fucose (F; red triangle), galactose (H; yellow circle), mannose (H; green circle), N-acetylneuraminic acid (S; purple diamond). The glycan MS data was normalized by calculating the relative abundance of each glycan as a fraction of the total detected glycans in each sample (min-max normalization). This approach was chosen to allow comparisons between samples without relying on absolute glycan quantification, which may vary due to differences in sample preparation or instrument sensitivity.

1.6. Corona Isolation and Washing Protocol

The protocol used in this study was previously applied in Trinh et al.[11], where one-wash corona samples were enriched with fibrinogen, while multiple washes resulted in similar corona fingerprints but with attenuated signal intensity. The same methodology was used here to ensure comparability with prior studies.

To evaluate the effect of washing steps on the corona composition, we analyzed hard corona (HC) samples subjected to three washes and soft corona (SC) samples processed with a single wash. As shown in Figure S1, both conditions resulted in similar corona fingerprints, with the HC samples displaying a reduced signal intensity. Based on these observations, SC was selected for further analyses due to its higher signal intensity and improved time efficiency.

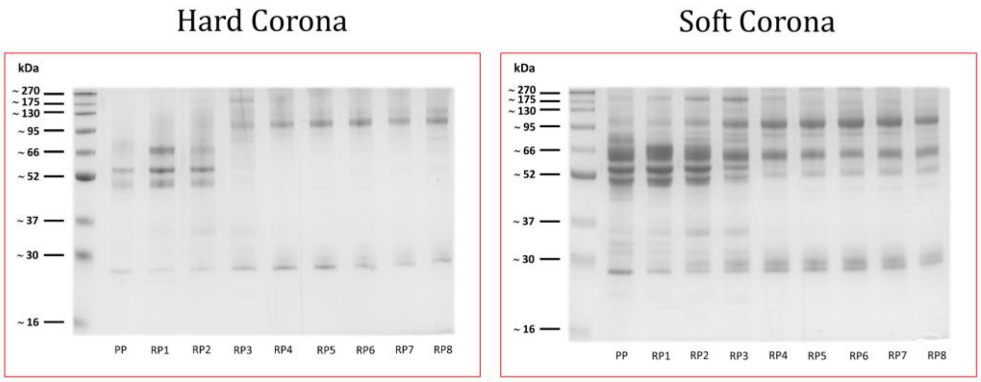

Figure S1. SDS-PAGE analysis of protein corona composition after different washing steps. The comparison between HC with three washes and SC with a single wash shows that the overall corona fingerprint remains consistent, with a decrease in signal intensity for HC samples.

It has to be pointed out that, to minimize background contamination, following an established protocol.[1] However, in order to determine the potential contribution of background protein adsorption to the observed corona profiles, we examined nanoparticle-free controls under identical experimental conditions. The resulting protein adsorption profile on microcentrifuge tube walls was analyzed after centrifugation (Figure S2). After PBS resuspension, the protein signal was notably reduced, indicating that background adsorption had a minimal influence on the observed corona composition.

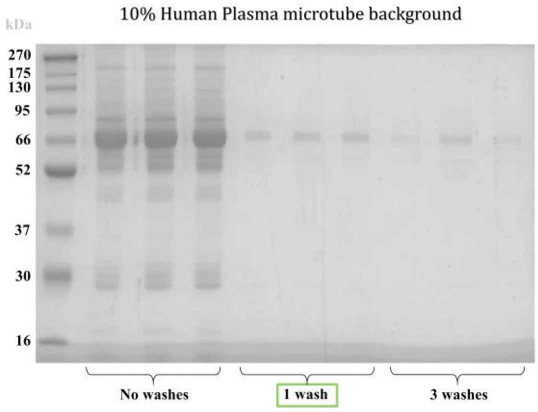

Figure S2. SDS-PAGE analysis of protein adsorption to microcentrifuge tubes in nanoparticle-free controls. Protein profiles were evaluated under different washing conditions to assess potential background contributions to the corona composition. The signal is reduced after PBS resuspension, indicating minimal interference from tube adsorption.

Moreover, Figure S3 shows that the background contribution is negligible compared to the signals of the proteins adsorbed onto the NP.

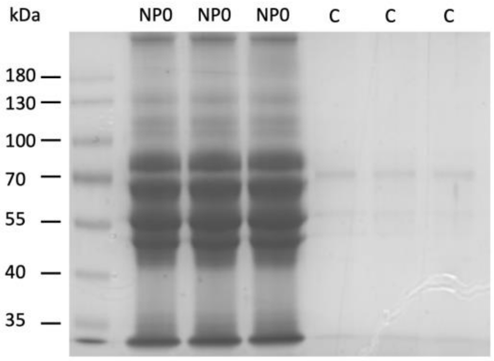

Figure S3. SDS-PAGE analysis of the protein background in relation to the NP protein corona (NP0). NP0 complexes were obtained following one single exposure to the biological fluid followed by one wash, as detailed in the material and methods section. A control sample was run for comparison, where the blood plasma was processed in the same way as in the NP0 sample but without the addition of NPs, like in Figure S2.

1.7. Effect of Single vs. Sequential Nanoparticle Exposures

To investigate whether the results on fluid fractionation of the sequential NP exposure process could be replicated by a single high-dose NP incubation, we conducted an additional experiment in which 4 mg of nanoparticles (equivalent to the total NP mass used across eight sequential exposures) were added to a 10% plasma solution in a single step. The resulting soft corona (SC) and hard corona (HC) compositions are shown in Figure S4.

The results indicate that while the corona composition remains distinct from that of blood plasma, the sequential evolution of the corona is lost when all NPs are added at once. This suggests that the progressive removal of corona proteins is necessary to achieve the gradually tuned corona composition observed after multiple exposures and washes. This observation is reflected in the manuscript, where the process is generalized to *n* exposure cycles to describe its broader applicability.

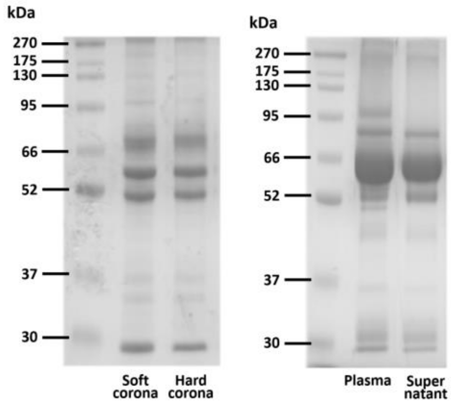

Figure S4. SDS-PAGE analysis showing the results for a single high-dose NP incubation, showing NP-corona proteins at the left and fluid proteins at the right. As observed, the fluid is fractionated, despite the sequential tuning of the NP corona is no longer possible.

2. SUPPLEMENTARY DATA

Table S1. Protein Mass Spectrometry

The data is openly available in Zenodo at https://doi.org/10.5281/zenodo.15050700.

Table S2. Glycan Assignments

The data is openly available in Zenodo at https://doi.org/10.5281/zenodo.15050700.

Table S3. Glycan Mass Spectrometry

The data is openly available in Zenodo at https://doi.org/10.5281/zenodo.15050700.

Table S4. Glycan Notation

Glycan composition, Oxford notation and Depiction Examples of the N-glycan structures described in this article. The glycan linkages and the position of fucoses were not specified.

Glycan composition	Oxford notation	Depiction Example (SNFG notation)
H3N3F1	FA1	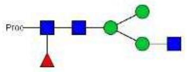
H3N4	A2	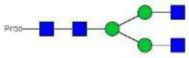
H3N4F1	FA2	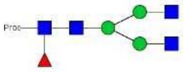
H3N5	A2B	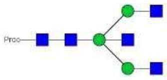
H3N5F1	FA2B	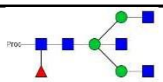
H4N3	A1G1	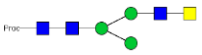
H4N3F1	FA1G1	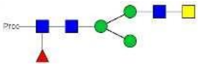

H4N3F1S1	FA1G1S1	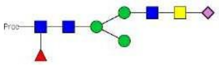
H4N3S1	A1G1S1	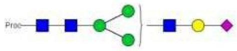
H4N4	A2G1	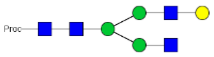
H4N4F1	FA2G1	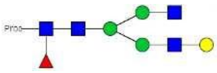
H4N4F1S1	FA2G1S1	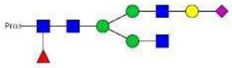
H4N4S1	A2G1S1	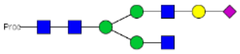
H4N5	A2BG1	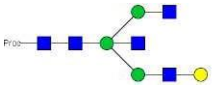
H4N5F1	FA2BG1	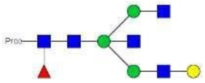
H4N5F1S1	FA2BG1S1	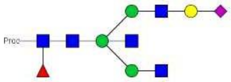
H4N5S1	A2BG1S1	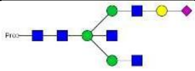
H5N2	M5	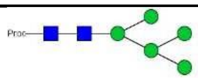
H5N3	M4A1G1	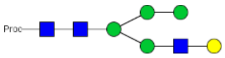
H5N3S1	M4A1G1S1	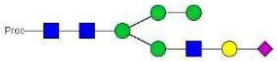
H5N4	A2G2	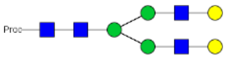
H5N4F1	FA2G2	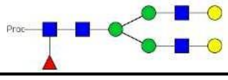
H5N4F1S1	FA2G2S1	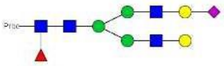
H5N4F1S2	FA2G2S2	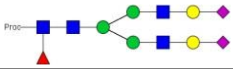
H5N4F2S1	FA2F1G2S1	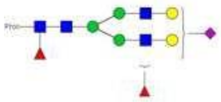
H5N4S1	A2G2S1	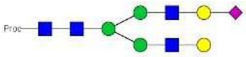
H5N4S2	A2G2S2	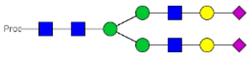
H5N5	A2BG2	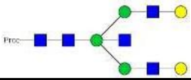
H5N5F1	FA2BG2	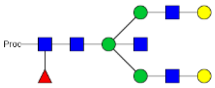

H5N5F1S1	FA2BG2S1	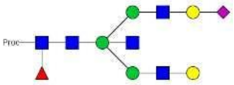
H5N5F1S2	FA2BG2S2	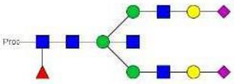
H5N5S1	A2BG2S1	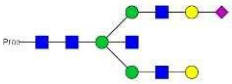
H5N5S2	A2BG2S2	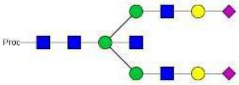
H6N2	M6	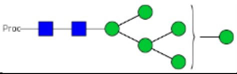
H6N3	M5A1G1	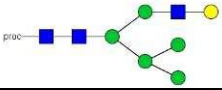
H6N3S1	M5A1G1S1	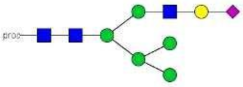
H6N4S1	M5BA1G1S1	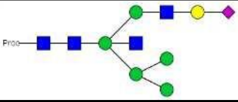
H6N5F1S1	A3F1G3S1	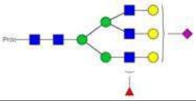
H6N5F1S2	A3F1G3S2	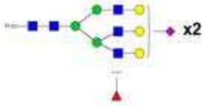
H6N5F1S3	A3F1G3S3	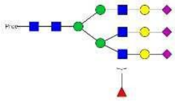
H6N5F2S3	A3F2G3S3	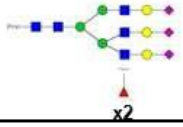
H6N5S1	A3G3S1	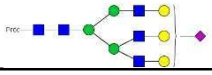
H6N5S2	A3G3S2	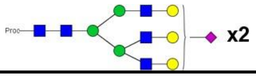
H6N5S3	A3G3S3	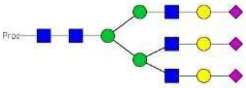
H7N2	M7	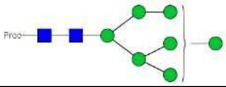
H7N6S1	A4G4S1	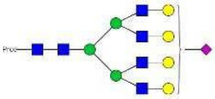
H7N6S2	A4G4S2	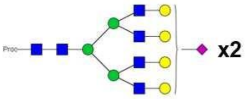

H7N6S3	A4G4S3	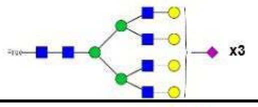
H7N6S4	A4G4S4	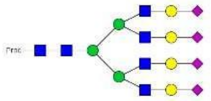
H7N6F1S3	A4F1G4S3	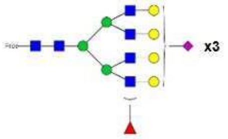
H7N6F1S4	A4F1G4S4	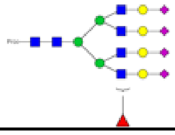
H8N2	M8	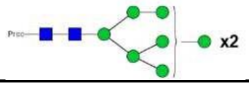
H9N2	M9	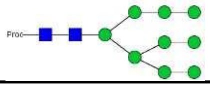

REFERENCES

1. Soliman, M.; Martinez-Serra, A.; Dobricic, M.; Trinh, D. N.; Cheeseman, J.; Spencer, D.; Monopoli, M. P., Standard protocols for isolation and characterization of nanoparticle biomolecular corona complexes. *Frontiers in Toxicology* **2024,** *6*, 1393330.

2. Cox, J.; Neuhauser, N.; Michalski, A.; Scheltema, R. A.; Olsen, J. V.; Mann, M., Andromeda: a peptide search engine integrated into the MaxQuant environment. *Journal of proteome research* **2011,** *10* (4), 1794-1805.

3. Cox, J.; Mann, M., MaxQuant enables high peptide identification rates, individualized ppb-range mass accuracies and proteome-wide protein quantification. *Nature biotechnology* **2008,** *26* (12), 1367-1372.

4. Tyanova, S.; Temu, T.; Sinitcyn, P.; Carlson, A.; Hein, M. Y.; Geiger, T.; Mann, M.; Cox, J., The Perseus computational platform for comprehensive analysis of (prote) omics data. *Nature methods* **2016,** *13* (9), 731-740.

5. Karpievitch, Y. V.; Dabney, A. R.; Smith, R. D., Normalization and missing value imputation for label-free LC-MS analysis. *BMC bioinformatics* **2012,** *13*, 1-9.

6. Jansen, B. C.; Hafkenscheid, L.; Bondt, A.; Gardner, R. A.; Hendel, J. L.; Wuhrer, M.; Spencer, D. I. R., HappyTools: a software for high-throughput HPLC data processing and quantitation. *PloS one* **2018,** *13* (7), e0200280.

7. Pino, L. K.; Searle, B. C.; Bollinger, J. G.; Nunn, B.; MacLean, B.; MacCoss, M. J., The Skyline ecosystem: Informatics for quantitative mass spectrometry proteomics. *Mass spectrometry reviews* **2020,** *39* (3), 229-244.

8. Saldova, R.; Asadi Shehni, A.; Haakensen, V. D.; Steinfeld, I.; Hilliard, M.; Kifer, I.; Helland, Å.; Yakhini, Z.; Børresen-Dale, A.-L.; Rudd, P. M., Association of N-glycosylation with breast carcinoma and systemic features using high-resolution quantitative UPLC. *Journal of proteome research* **2014,** *13* (5), 2314-2327.

9. Shubhakar, A.; Jansen, B. C.; Adams, A. T.; Reiding, K. R.; Ventham, N. T.; Kalla, R.; Bergemalm, D.; Urbanowicz, P. A.; Gardner, R. A.; IBD-BIOM Consortium; Wuhrer, M.; Halfvarson, J.; Satsangi, J.; Fernandes, D. L.; Spencer, D. I. R., Serum N-glycomic biomarkers predict treatment escalation in inflammatory bowel disease. *Journal of Crohn's and Colitis* **2023,** *17* (6), 919-932.

10. Varki, A.; Cummings, R. D.; Aebi, M.; Packer, N. H.; Seeberger, P. H.; Esko, J. D.; Stanley, P.; Hart, G.; Darvill, A.; Kinoshita, T., Symbol nomenclature for graphical representations of glycans. *Glycobiology* **2015,** *25* (12), 1323-1324.

11. Trinh, D. N.; Gardner, R. A.; Franciosi, A. N.; McCarthy, C.; Keane, M. P.; Soliman, M. G.; O'Donnell, J. S.; Meleady, P.; Spencer, D. I.; Monopoli, M. P., Nanoparticle biomolecular corona-based enrichment of plasma glycoproteins for N-glycan profiling and application in biomarker discovery. *ACS nano* **2022,** *16* (4), 5463-5475.
